# Supplementary material for: The contributions of cartilage endplate composition and vertebral bone marrow fat to intervertebral disc degeneration in patients with chronic low back pain
Source: Eur Spine J. 2022 Apr 20;31(7):1866–72. doi: 10.1007/s00586-022-07206-x (PMC9252939; doi:10.1007/s00586-022-07206-x)
Supplement: Supplementary file 1 — Supplementary file1 (DOCX 23 KB) [file 586_2022_7206_MOESM1_ESM.docx]

**Contributions of cartilage endplate composition and vertebral bone marrow fat to intervertebral disc degeneration in patients with chronic low back pain**

Noah B. Bonnheim, PhD; Linshanshan Wang; Ann A. Lazar, PhD; Jiamin Zhou; Ravi Chachad; Nico Sollmann, MD, PhD; Xiaojie Guo, MPH; Claudia Iriondo, PhD; Conor O’Neill, MD; Jeffery C. Lotz, PhD; Thomas M. Link, MD, PhD; Roland Krug, PhD; Aaron J. Fields, PhD^[[1]](#footnote-1)^

Supplemental Material

*Detailed methods*

MRI of the lumbar spine was performed on a GE 3T Discovery MR750 scanner using an 8-channel phased-array spine coil (GE Healthcare, Waukesha, WI). MRI data used for the purposes of this study were acquired using sagittal acquisitions from a multi-echo UTE Cones sequence, a chemical shift encoding-based water-fat sequence, a T1ρ mapping sequence, and standard clinical fast spin-echo sequences with T_1_- and T_2_-weighting. The sequence details are as follows:

1. Multi-echo 3D UTE Cones sequence: echo time (TE) = 0.24, 5.2, 10.2, 15.2, 20.2, 25.2 ms; repetition time (TR) = 32 ms; field-of-view (FOV) = 28 cm; flip angle = 15°; in-plane resolution = 0.5 mm; slice-thickness = 3 mm (interpolated to 1 mm).
2. Six-echo water-fat sequence consisting of a 3D spoiled gradient-recalled echo (SPGR) sequence with iterative decomposition of water and fat with echo asymmetry and least-squares estimation (IDEAL) reconstruction: TE = 2, 3, 4, 5, 6, 7 ms; TR = 6.2 ms; FOV = 26 cm; flip angle = 3°; in-plane resolution = 1.3 mm; slice thickness = 4 mm, receiver bandwidth = 83.3 kHz.
3. 3D T1ρ mapping sequence consisting of a magnetization‐prepared angle‐modulated partitioned k‐space SPGR sequence: spin-lock time (TSL) = 0, 10, 40, and 80 ms; TR = 5.2 ms; FOV = 20 cm; flip angle = 60°; in-plane resolution = 0.78 mm ; slice thickness = 8 mm; spin-lock frequency = 300 Hz.
4. Standard clinical fast spin-echo with T_1_- and T_2_-weighting: TE = 15, 60 ms (T_1_-, T_2_-weighted, respectively); TR = 511, 4877 ms; echo train length (ETL) = 24, 4; FOV = 26 cm; in-plane resolution = 0.5 mm; slice thickness = 3 mm sagittal, 4 mm axial; receiver bandwidth = 50–62.5 kHz.

1. Corresponding author. University of California, San Francisco. [aaron.fields@ucsf.edu](mailto:aaron.fields@ucsf.edu) [↑](#footnote-ref-1)
